# Enhanced energy-constrained quantum communication over bosonic Gaussian channels

**DOI:** 10.1038/s41467-020-14329-6

**Published:** 2020-01-23

**Authors:** Kyungjoo Noh, Stefano Pirandola, Liang Jiang

**Affiliations:** 1 Departments of Applied Physics and Physics, Yale University, New Haven, CT 06511 USA; 2 Yale Quantum Institute, Yale University, New Haven, CT 06520 USA; 3 Computer Science and York Centre for Quantum Technologies, University of York, York, YO10 5GH UK; 4 Research Laboratory of Electronics, Massachusetts Institute of Technology (MIT), Cambridge, MA 02139 USA; 5 Pritzker School of Molecular Engineering, University of Chicago, 5640 South Ellis Avenue, Chicago, IL 60637 USA

**Keywords:** Fibre optics and optical communications, Quantum physics, Quantum information, Theoretical physics

## Abstract

Quantum communication is an important branch of quantum information science, promising unconditional security to classical communication and providing the building block of a future large-scale quantum network. Noise in realistic quantum communication channels imposes fundamental limits on the communication rates of various quantum communication tasks. It is therefore crucial to identify or bound the quantum capacities of a quantum channel. Here, we consider Gaussian channels that model energy loss and thermal noise errors in realistic optical and microwave communication channels and study their various quantum capacities in the energy-constrained scenario. We provide improved lower bounds to various energy-constrained quantum capacities of these fundamental channels and show that higher communication rates can be attained than previously believed. Specifically, we show that one can boost the transmission rates of quantum information and private classical information by using a correlated multi-mode thermal state instead of the single-mode thermal state of the same energy.

## Abstract (web summary)

The amount of information that a quantum channel can transmit is fundamentally bounded by the amount of noise in the channel. Here, the authors consider the realistic case with loss and thermal noise errors and prove that correlated multi-mode thermal states can achieve higher rates than single-mode ones.

## Introduction

Quantum communication is a field of quantum information science that takes advantage of unique quantum mechanical nature of information carriers to realize secure classical communications^1,2^ and build a large-scale quantum network^3–5^. Realistic quantum communication channels are noisy and therefore quantum error correction or entanglement distillation^6–8^ is essential to faithfully implement various quantum communication tasks. Due to the resource overhead associated with the error correction or entanglement distillation, quantum communication rates are fundamentally limited by the noise in quantum channels that are used to transmit quantum information. Thus, determining the maximum achievable quantum communication rates of an experimentally relevant noisy quantum channel is of fundamental importance to the quantum information science.

A celebrated result due to Shannon^9^ established that the maximum achievable (classical) communication rate of a (classical) channel equals the channel’s mutual information, or the channel’s (classical) capacity. The notion of (classical) channel capacity has been generalized to the quantum realm^10–12^, and there are various notions of quantum capacities that characterize the channel’s maximum achievable quantum communication rates for various quantum communication tasks (see, e.g., ref. ^12^ Sec. 8). For example, the regularized private information ${P}_{{\rm{reg}}}({\mathcal{N}})$ of a channel ${\mathcal{N}}$ quantifies the maximum achievable secure classical communication rate of the channel, also known as the channel’s private capacity^13^. Similarly, the regularized coherent information ${Q}_{{\rm{reg}}}({\mathcal{N}})$ quantifies the maximum achievable quantum state transmission rate (without classical feedback assistance), also known as the channel’s quantum capacity^13–17^.

Evaluation of these quantities, however, requires optimization over all input states to infinitely many channels^18–23^, and therefore is generally intractable^24,25^ unless the channel has a special structure such as degradability or anti-degradability^26–28^. Moreover, explicit formulas for the two-way quantum capacity (i.e., maximum quantum state transmission rate with two-way classical feedback assistance) are only known for so-called distillable channels^29^ i.e., channels whose Choi matrices have relative entropy of entanglement^30–32^ equal to the one-way distillable entanglement. Even though the two-way quantum (and private) capacities of these channels are known^29^ and also have suitable generalizations to repeater chains and quantum networks of arbitrary topology^33,34^, finding similar results for other types of quantum channels is the subject of intensive investigation, especially within the setting of continuous variable systems^35^.

In this work, we consider bosonic Gaussian channels^36^ and study their various quantum capacities in the energy-constrained scenario^37^. Among Gaussian channels, thermal-loss channels characterize energy loss and thermal-noise errors and thus model realistic optical and microwave quantum communication channels, i.e., two leading platforms for quantum communication technologies. Therefore, understanding the quantum capacities of Gaussian channels is of great practical importance, as well as of academic interest. Previously, it was shown that bosonic pure-loss channels (a subclass of thermal-loss channels that do not have thermal noise) are either degradable or anti-degradable^27^. Thus, various quantum capacities of these subclass of Gaussian channels are well understood and determined analytically^29,38,39^.

In practice, there is additional thermal noise added to the communication channels, which can be induced by the laser noise in optical communication or by the background thermal noise in microwave communication^40,41^. Hence, it is important to understand the quantum channel capacities for these more general thermal-loss channels. However, thermal-loss channels are neither degradable nor anti-degradable^42,43^ and only lower^38,44–47^, and upper bounds^29,38,47–49^ are known for their various quantum and private capacities (one-way, two-way, energy-constrained or unconstrained).

In this paper, we establish improved lower bounds of various energy-constrained quantum capacities of thermal-loss channels that are stronger than the existing bounds. That is, we show that higher quantum communication rates can be achieved than previously believed. Specifically, we construct a family of multimode Gaussian states, called correlated multimode thermal states, and show that they yield larger coherent information (per channel use) than the corresponding single-mode thermal state of the same energy in the low input energy regime. We also show that higher two-way quantum communication rates can be achieved by using correlated multimode thermal states and hybridizing forward and backward strategies, instead of using single-mode thermal states and exclusively using a forward or a backward strategy. Finally, we apply a similar technique to further improve the lower bound of the energy-constrained private capacity of the thermal-loss channel.

## Results

### Correlated multimode thermal states

We first construct a family of Gaussian multimode states, called correlated multimode thermal states, which is the key ingredient for improving the lower bounds of various quantum capacities of Gaussian channels (see ref. ^36^ or the Methods section for the definition of Gaussian states and channels). Let $\hat{\tau }(\bar{n})$ denote the single-mode thermal state with an average photon number ${\rm{Tr}}[\hat{n}\hat{\tau }(\bar{n})]=\bar{n}$, that is $\hat{\tau }(\bar{n})\equiv \sum _{n=0}^{\infty }\frac{{\bar{n}}^{n}}{{(1+\bar{n})}^{n+1}}\left|n\right\rangle \left\langle n\right|$, where *n* is a Fock state. Uncorrelated multimode thermal states would then simply be given by a tensor product of single-mode thermal states ${\left\{\hat{\tau }(\bar{n})\right\}}^{\otimes N}$. Now, we define correlated multimode thermal states as follows:

1 $$\hat{{\mathcal{T}}}({\bf{N}},{\bf{n}})\equiv {\hat{U}}_{{\rm{GFT}}}^{(N)}\left[{\left\{\hat{\tau }({\bar{n}}_{1})\right\}}^{\otimes {N}_{1}}\otimes \cdots \otimes {\left\{\hat{\tau }({\bar{n}}_{r})\right\}}^{\otimes {N}_{r}}\right]{\left({\hat{U}}_{{\rm{GFT}}}^{(N)}\right)}^{\dagger }.$$

**Fig. 1:**
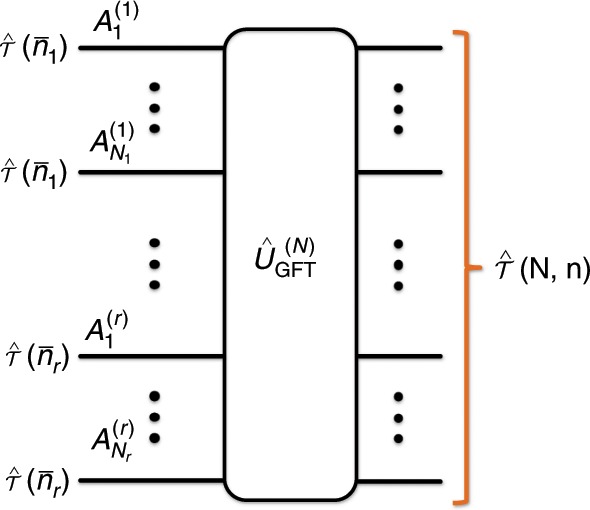
Generation of a correlated multimode thermal state. A correlated multimode thermal state $\hat{{\mathcal{T}}}({\bf{N}},{\bf{n}})$ with **N** = (*N*_1_, ⋯ , *N*_*r*_) and ${\bf{n}}=({\bar{n}}_{1},\cdots \ ,{\bar{n}}_{r})$ (such that $\sum _{k=1}^{r}{N}_{k}=N$) can be generated by applying the *N*-mode Gaussian Fourier transformation ${\hat{U}}_{{\rm{GFT}}}^{(N)}$ to an uncorrelated thermal state ${\left\{\hat{\tau }({\bar{n}}_{1})\right\}}^{\otimes {N}_{1}}\otimes \cdots \otimes {\left\{\hat{\tau }({\bar{n}}_{r})\right\}}^{\otimes {N}_{r}}$.

Here, **N** = (${N_1}, \, \cdots \, ,{N_r}$) such that $\sum _{k=1}^{r}{N}_{k}=N$ and ${\bf{n}}=({\bar{n}}_{1},\cdots \ ,{\bar{n}}_{r})$. ${\hat{U}}_{{\rm{GFT}}}^{(N)}$ is the *N*-mode Gaussian Fourier transformation whose action on the *j*th annihilation operator ${\hat{a}}_{j}$ is defined as follows:

2 $${\left({\hat{U}}_{{\rm{GFT}}}^{(N)}\right)}^{\dagger }{\hat{a}}_{j}{\hat{U}}_{{\rm{GFT}}}^{(N)}=\frac{1}{\sqrt{N}}\sum _{k=1}^{N}{e}^{i\frac{2\pi }{N}(j-1)(k-1)}{\hat{a}}_{k}$$

which holds for all *j* ∈ {$1, \cdots \, ,N$}. Hence, the correlated multimode thermal state $\hat{{\mathcal{T}}}({\bf{N}},{\bf{n}})$ is a collection of single-mode thermal states (where each of the first *N*_1_ modes supports on average ${\bar{n}}_{1}$ photons, each of the next *N*_2_ modes supports on average ${\bar{n}}_{2}$ photons and so on), which are uniformly mixed by the Gaussian Fourier transformation ${\hat{U}}_{{\rm{GFT}}}^{(N)}$ (see Fig. 1). We remark that each mode in the correlated *N*-mode thermal state $\hat{{\mathcal{T}}}({\bf{N}},{\bf{n}})$ supports on average $\bar{n}=\frac{1}{N}\sum _{k=1}^{r}{N}_{k}{\bar{n}}_{k}$ photons.

A simple nontrivial example of correlated multimode thermal states would be $\hat{{\mathcal{T}}}({\bf{N}},{\bf{n}})$ with **N** = (1, *N* − 1) and ${\bf{n}}=(N\bar{n},0)$, and its covariance matrix^36^ is given by

3 $$V=\left[\begin{array}{llll}(\bar{n}+\frac{1}{2}){I}_{2}&\bar{n}{I}_{2}&\cdots \ &\bar{n}{I}_{2}\\ \bar{n}{I}_{2}&(\bar{n}+\frac{1}{2}){I}_{2}&\cdots \ &\bar{n}{I}_{2}\\ \vdots &\vdots &\ddots &\vdots \\ \bar{n}{I}_{2}&\bar{n}{I}_{2}&\cdots \ &(\bar{n}+\frac{1}{2}){I}_{2}\end{array}\right],$$

where *I*_2_ is the 2 × 2 identity matrix. As can be seen from the diagonal elements of the covariance matrix, every mode supports on average $\bar{n}$ photons. Therefore, the reduced density matrix of each mode is given by a single-mode thermal state $\hat{\tau }(\bar{n})$. On the other hand, the off-diagonal elements of the covariance matrix indicate that the position (or the momentum) quadratures of every pair of modes are positively correlated: this is what distinguishes $\hat{{\mathcal{T}}}({\bf{N}},{\bf{n}})$ from the uncorrelated *N*-mode thermal state ${\left\{\hat{\tau }(\bar{n})\right\}}^{\otimes N}$ and why we call it a correlated multimode thermal state.

We remark that correlated multimode thermal states can be efficiently prepared because the Gaussian Fourier transformation ${\hat{U}}_{{\rm{GFT}}}^{(N)}$ can be implemented efficiently by using a variant of the fast Fourier transform technique^50^.

### Coherent information and quantum capacity

Let ${\mathcal{N}}$ be a quantum channel and ${I}_{{\rm{c}}}({\mathcal{N}},\hat{\rho })$ denote the channel’s coherent information with respect to an input state $\hat{\rho }$, i.e.,

4 $${I}_{{\rm{c}}}({\mathcal{N}},\hat{\rho })\equiv S\left({\mathcal{N}}(\hat{\rho })\right)-S\left({{\mathcal{N}}}^{{\rm{c}}}(\hat{\rho })\right),$$

where $S(\hat{\rho })\equiv -{\rm{Tr}}[\hat{\rho }\,{{\mathrm{log}}}_{2} \, \hat{\rho}]$ is the quantum von Neumann entropy of a state $\hat{\rho }$ and ${{\mathcal{N}}}^{{\rm{c}}}$ is the complementary channel of ${\mathcal{N}}$. The quantum capacity ${C}_{{\rm{Q}}}({\mathcal{N}})$ of the channel ${\mathcal{N}}$ (i.e., the maximum achievable quantum state transmission rate without classical feedback assistance) is equal to the channel’s regularized coherent information ${Q}_{{\rm{reg}}}({\mathcal{N}})$^13–17^:

5 $${C}_{{\rm{Q}}}({\mathcal{N}})={Q}_{{\rm{reg}}}({\mathcal{N}})\equiv \mathop{{\mathrm{lim}}}\limits_{N\to \infty }\frac{1}{N}\mathop{{\mathrm{max}}}\limits_{\hat{\rho }}{I}_{{\rm{c}}}({{\mathcal{N}}}^{\otimes N},\hat{\rho }).$$

In the energy-constrained case, the coherent information should be optimized over all input states that satisfy an energy-constraint, such that at most $\bar{n}$ mean photons are fed to the channel in each use.

The bosonic pure-loss channel ${\mathcal{N}}[\eta ,0]$ with a transmissivity *η* ∈ [0, 1] (or loss probability *γ* ≡ 1 − *η*) is either degradable (*η* ∈ (1∕2, 1]) or anti-degradable (*η* ∈ [0, 1∕2])^27^. Therefore, the regularization of its coherent information is unnecessary^26–28^, and the optimal input state subject to an average photon number constraint ${\rm{Tr}}[{\hat{n}}_{k}\hat{\rho }]\ \le \ \bar{n}$ for all *k* ∈ {1, ⋯ , *N*} is shown to be the single-mode thermal state $\hat{\tau }(\bar{n})$^39,51^ (see also ref. ^49^):

6 $${C}_{{\rm{Q}}}^{\le \bar{n}}({\mathcal{N}}[\eta ,0])=	 \mathop{{\mathrm{lim}}}\limits_{N\to \infty }\frac{1}{N}\mathop{{\mathrm{max}}}\limits_{\hat{\rho }:{\rm{Tr}}[{\hat{n}}_{k}\hat{\rho }]\le \bar{n}\forall k}{I}_{{\rm{c}}}({\mathcal{N}}{[\eta ,0]}^{\otimes N},\hat{\rho })\\ = 	\hskip 2.5pt {I}_{{\rm{c}}}({\mathcal{N}}[\eta ,0],\hat{\tau }(\bar{n}))=g(\eta \bar{n})-g((1-\eta )\bar{n}),$$

where $g(x)\equiv S(\hat{\tau }(x))=(x+1) \, {{\mathrm{log}}}_{2}(x+1)-x \, {{\mathrm{log}}}_{2} \, x$ is the entropy of the thermal state $\hat{\tau }(x)$^52^.

On the other hand, a general thermal-loss channel ${\mathcal{N}}[\eta ,{\bar{n}}_{{\rm{th}}}]$ with a nonzero environmental thermal photon number ${\bar{n}}_{{\rm{th}}} \, \ne \, 0$ is neither degradable nor anti-degradable^42,43^. In this case, the single-mode thermal state $\hat{\tau }(\bar{n})$ is not necessarily the optimal input state, and the associated coherent information (evaluated in ref. ^38^) only lower bounds the quantum capacity, i.e.,

7 $${C}_{{\rm{Q}}}^{\le \bar{n}}({\mathcal{N}}[\eta ,{\bar{n}}_{{\rm{th}}}])\ge	 \, {I}_{{\rm{c}}}({\mathcal{N}}[\eta ,{\bar{n}}_{{\rm{th}}}],\hat{\tau }(\bar{n}))\\ =	 \, g(\eta \bar{n}+(1-\eta ){\bar{n}}_{{\rm{th}}})\\ 	-g\left(\frac{D+(1-\eta )(\bar{n}-{\bar{n}}_{{\rm{th}}})-1}{2}\right)\\ 	-g\left(\frac{D-(1-\eta )(\bar{n}-{\bar{n}}_{{\rm{th}}})-1}{2}\right),$$

where $D\equiv \sqrt{{((1+\eta )\bar{n}+(1-\eta ){\bar{n}}_{{\rm{th}}}+1)}^{2}-4\eta \bar{n}(\bar{n}+1)}$. This is the best-known lower bound for ${C}_{{\rm{Q}}}({\mathcal{N}}[\eta ,{\bar{n}}_{{\rm{th}}}])$ to date before our work. Below, we demonstrate that correlated multimode thermal states can outperform the single-mode thermal state of the same energy in the noisy channel (near-zero capacity) regime. By doing so, we show that higher quantum state transmission rates can be attained for thermal-loss channels than previously believed.

**Theorem 1** *Consider a correlated* *N*-*mode thermal state* $\hat{{\mathcal{T}}}({\bf{N}},{\bf{n}})$ *with* **N** = (*M*, *N* − *M*) *and* ${\bf{n}}=(\frac{N}{M}\bar{n},0)$ *and let* $x=\frac{M}{N}$, *where* *M* ∈ {1, ⋯ , *N*}. *Then, the coherent information with respect to the input state* $\hat{{\mathcal{T}}}({\bf{N}},{\bf{n}})$ *is given by*

8 $$\frac{1}{N}{I}_{{\rm{c}}}\left({\mathcal{N}}{[\eta ,{\bar{n}}_{{\rm{th}}}]}^{\otimes N},\hat{{\mathcal{T}}}({\bf{N}},{\bf{n}})\right)=x{I}_{{\rm{c}}}\left({\mathcal{N}}[\eta ,{\bar{n}}_{{\rm{th}}}],\hat{\tau }\left(\frac{\bar{n}}{x}\right)\right).$$

Since *x* can be any rational number in (0, 1] and the set of rational numbers is a dense subset of the set of real numbers, we have the following improved lower bound of the quantum capacity of thermal-loss channels.

9 $${C}_{{\rm{Q}}}^{\le \bar{n}}({\mathcal{N}}[\eta ,{\bar{n}}_{{\rm{th}}}])\ge \mathop{{\mathrm{max}}}\limits_{0<x\le 1}x{I}_{{\rm{c}}}\left({\mathcal{N}}[\eta ,{\bar{n}}_{{\rm{th}}}],\hat{\tau }\left(\frac{\bar{n}}{x}\right)\right).$$

The proof of Theorem 1 is given in the Methods section. Note that for **N** = (*M*, *N* − *M*) and ${\bf{n}}=(\frac{N}{M}\bar{n},0)$, ${\rm{Tr}}[{\hat{n}}_{k}\hat{{\mathcal{T}}}({\bf{N}},{\bf{n}})]=\bar{n}$ holds for all *k* ∈ {1, ⋯ , *N*}, and thus $\hat{{\mathcal{T}}}({\bf{N}},{\bf{n}})$ is a valid input state that satisfies the energy constraint. Also, our new bound in Eq. (9) is at least as tight as the previous bound in Eq. (7), since the previous bound can be recovered by plugging in *x* = 1 to the objective function. Below, we show that in the noisy channel (near-zero capacity) regime, the optimal value of *x* can be strictly <1, and thus our bound is strictly tighter than the previous bound. We also explain this behavior in an intuitive manner by using simple mathematical concepts such as convexity of a function and convex hull of a non-convex region.

**Fig. 2:**
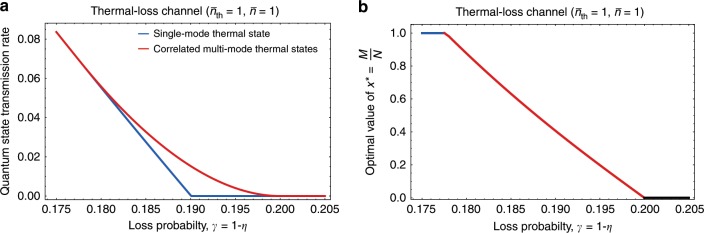
Achievable quantum state transmission rate of thermal-loss channels. **a** Quantum state transmission rate of thermal-loss channels ${\mathcal{N}}[\eta ,{\bar{n}}_{{\rm{th}}}=1]$ as a function of the loss probability *γ* = 1 − *η* achievable with the single-mode thermal state $\hat{\tau }(\bar{n})$ (blue, Eq. (7)), and with a correlated multimode thermal state $\hat{{\mathcal{T}}}({\bf{N}},{\bf{n}})$ (red, Eq. (9)) subject to the maximum allowed average photon number $\bar{n}=1$ per channel use. For the correlated multimode thermal states, the achievable rate was evaluated by taking $M/N={x}^{\star }={{\rm{argmax}}}_{0<x\le 1}x{I}_{{\rm{c}}}({\mathcal{N}}[\eta ,{\bar{n}}_{{\rm{th}}}],\hat{\tau }(\bar{n}/x))$, where **N** = (*M*, *N* − *M*) and ${\bf{n}}=(\frac{N}{M}\bar{n},0)$. **b** The optimal value of ${x}^{\star }$ = *M*∕*N* at each *γ*, color-coded by the type of optimizer (blue: single-mode thermal states; red: correlated multimode thermal states), that yields the maximum quantum state transmission rate. We set ${x}^{\star }$ = *M*∕*N* = 0, when all the states we consider yield vanishing quantum state transmission rate (black).

To demonstrate that our new bound can be strictly tighter than the previous bound, we take a family of thermal-loss channels ${\mathcal{N}}[\eta ,{\bar{n}}_{{\rm{th}}}]$ with ${\bar{n}}_{{\rm{th}}}=1$ and compute the new bound in Eq. (9) for each *η* = 1 − *γ*, assuming that the maximum allowed average photon number per channel is $\bar{n}=1$. In Fig. 2a, we plot the quantum state transmission rates achievable with the single-mode thermal state $\hat{\tau }(\bar{n}=1)$ and with the correlated multimode thermal states $\hat{{\mathcal{T}}}({\bf{N}},{\bf{n}})$. When the loss probability is low (i.e., *γ* ≤ 0.1775), the single-mode thermal state yields the largest coherent information. However, when the loss probability is higher (*γ* ≥ 0.1775), there exists a correlated multimode thermal state that outperforms the single-mode thermal state. Thus, we established a tighter lower bound to the quantum capacity of thermal-loss channels than previously known^38^. In Fig. 2b, we plot the optimal value of *M*∕*N* as a function of *γ* that allows such a higher communication rate. It is important to note that only a finite number of modes is required if the optimal value of *x* is a rational number. For example, ${x}^{\star }$ = 3∕8 corresponds to the correlated eight-mode thermal state $\hat{{\mathcal{T}}}({\bf{N}},{\bf{n}})$ with **N** = (*M*, *N* − *M*) = (3, 5) and ${\bf{n}}=(8\bar{n}/3,0)$. On the other hand, if ${x}^{\star }$ is irrational, one needs infinitely many modes to accurately obtain the rate $x{I}_{{\rm{c}}}({\mathcal{N}}[\eta ,{\bar{n}}_{{\rm{th}}}],\hat{\tau }(\bar{n}/x)){| }_{x={x}^{\star }}$.

### Convexity of coherent information and superadditivity

**Fig. 3:**
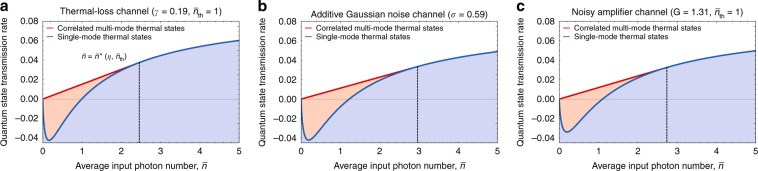
Convexity of coherent information and superadditivity. Achievable quantum state transmission rate of the single-mode (blue) and correlated multimode (red) thermal states as a function of $\bar{n}$ for (**a**) the thermal-loss channel ${\mathcal{N}}[\eta =0.81,{\bar{n}}_{{\rm{th}}}=1]$ **b** the additive Gaussian noise channel ${{\mathcal{N}}}_{{{\rm{B}}}_{2}}[\sigma =0.59]$ and **c** the noisy amplifier channel ${\mathcal{A}}[G=1.31,{\bar{n}}_{{\rm{th}}}=1]$. Note that in (**a**), the first-order contact point is given by ${\bar{n}}^{\star }(\eta =0.81,{\bar{n}}_{{\rm{th}}}=1)=2.458$, which corresponds to ${x}^{\star }$ = 1∕2.458 = 0.407. This value agrees with ${x}^{\star }$ = 0.407 which is independently obtained in Fig. 2b for *γ* = 0.19 and ${\bar{n}}_{{\rm{th}}}=1$ (see also the main text).

We now explain the nontrivial behavior shown in Fig. 2 (i.e., ${x}^{\star }$ < 1) in an intuitive way. Specifically, we relate the observed nontrivial behavior with the convexity of the coherent information ${I}_{{\rm{c}}}({\mathcal{N}}[\eta ,{\bar{n}}_{{\rm{th}}}],\hat{\tau }(\bar{n}))$ in the allowed average photon number $\bar{n}$ for fixed values of *η* and ${\bar{n}}_{{\rm{th}}}$. For concreteness, we take the thermal-loss channel ${\mathcal{N}}[\eta ,{\bar{n}}_{{\rm{th}}}]$ with *η* = 0.81 (or *γ* = 0.19) and ${\bar{n}}_{{\rm{th}}}=1$, and plot its coherent information ${I}_{{\rm{c}}}({\mathcal{N}}[\eta ,{\bar{n}}_{{\rm{th}}}],\hat{\tau }(\bar{n}))$ with respect to single-mode thermal states $\hat{\tau }(\bar{n})$ as a function of $\bar{n}$. As can be seen from the solid blue line in Fig. 3a, the coherent information ${I}_{{\rm{c}}}({\mathcal{N}}[\eta ,{\bar{n}}_{{\rm{th}}}],\hat{\tau }(\bar{n}))$ is convex in $\bar{n}$ for small $\bar{n}$ and concave for large $\bar{n}$. Consider the region of rates achievable by the single-mode thermal states ${A}_{\eta ,{\bar{n}}_{{\rm{th}}}}^{(1)}\equiv \{(\bar{n},R)| {\bar{n}}\ \ge \ 0\;{\rm{and}}\;R\ \le \ {I}_{{\rm{c}}}({\mathcal{N}}[\eta ,{\bar{n}}_{{\rm{th}}}],\hat{\tau }(\bar{n}))\}$ (shaded blue region in Fig. 3a) and also its convex hull ${A}_{\eta ,{\bar{n}}_{{\rm{th}}}}^{(\infty )}\equiv {\rm{ConvexHull}}({A}_{\eta ,{\bar{n}}_{{\rm{th}}}}^{(1)})$ (shaded red and blue regions in Fig. 3a). We observe that the region ${A}_{\eta ,{\bar{n}}_{{\rm{th}}}}^{(\infty )}$ is achievable by correlated multimode thermal states: consider a generic convex combination of *r* points in ${A}_{\eta ,{\bar{n}}_{{\rm{th}}}}^{(1)}$, i.e.,

10 $$\sum _{k=1}^{r}{\lambda }_{k}\left({\bar{n}}_{k},{I}_{{\rm{c}}}({\mathcal{N}}[\eta ,{\bar{n}}_{{\rm{th}}}],\hat{\tau }({\bar{n}}_{k}))\right),$$

where *λ*_*k*_ ≥ 0 for all *k* ∈ {1, ⋯ , *r*} and $\sum _{k=1}^{r}{\lambda }_{k}=1$. Then, the rate $\sum _{k=1}^{r}{\lambda }_{k}{I}_{{\rm{c}}}({\mathcal{N}}[\eta ,{\bar{n}}_{{\rm{th}}}],\hat{\tau }({\bar{n}}_{k}))$ can be achieved by a correlated multimode thermal state $\hat{{\mathcal{T}}}({\bf{N}},{\bf{n}})$ with **N** = (*N*_1_, ⋯ , *N*_*r*_) and ${\bf{n}}=({\bar{n}}_{1},\cdots \ ,{\bar{n}}_{r})$ such that *λ*_*k*_ = *N*_*k*_∕*N* for all *k* ∈ {1, ⋯ , *r*}, where $N=\sum _{k=1}^{r}{N}_{k}$. Note that *λ*_*k*_ should be a rational number. Similarly as above, however, by choosing a sufficiently large *N* one can approximate any irrational *λ*_*k*_ to a desired accuracy, which can be arbitrarily small.

Importantly, due to the convexity of the coherent information ${I}_{{\rm{c}}}({\mathcal{N}}[\eta ,{\bar{n}}_{{\rm{th}}}],\hat{\tau }(\bar{n}))$ in the small $\bar{n}$ regime, the region ${A}_{\eta ,{\bar{n}}_{{\rm{th}}}}^{(\infty )}$ properly contains the region ${A}_{\eta ,{\bar{n}}_{{\rm{th}}}}^{(1)}$, as indicated by the shaded red region in Fig. 3a. This is why correlated multimode thermal states outperform single-mode thermal states in the noisy channel regime. In particular, the highest achievable rate can be obtained by taking the convex combination of the origin (0, 0) and the first-order contact point (${\bar{n}}^{\star }(\eta ,{\bar{n}}_{{\rm{th}}}),{I}_{{\rm{c}}}({\mathcal{N}}[\eta ,{\bar{n}}_{{\rm{th}}}],\hat{\tau }({\bar{n}}^{\star }(\eta ,{\bar{n}}_{{\rm{th}}})))$ with some weights *λ* and 1 − *λ*, respectively (see the solid red line in Fig. 3). Note that the rate $x{I}_{{\rm{c}}}({\mathcal{N}}[\eta ,{\bar{n}}_{{\rm{th}}}],\hat{\tau }(\bar{n}/x))$ in Eq. (9) can be understood as the one that is derived from such a convex combination with $1-\lambda =x=\bar{n}/{\bar{n}}^{\star }(\eta ,{\bar{n}}_{{\rm{th}}})$. For example, in the case of thermal-loss channel ${\mathcal{N}}[\eta ,{\bar{n}}_{{\rm{th}}}]$ with *η* = 0.81 (or *γ* = 0.19) and ${\bar{n}}_{{\rm{th}}}=1$, the first-order contact point is given by ${\bar{n}}^{\star }(\eta ,{\bar{n}}_{{\rm{th}}})=2.458$ (see Fig. 3) which corresponds to *x* = 0.407 for $\bar{n}=1$: this agrees with the optimal value ${x}^{\star }$ = 0.407 in Fig. 2b for *η* = 0.81 (or *γ* = 0.19), ${\bar{n}}_{{\rm{th}}}=1$, and $\bar{n}=1$.

We remark that the coherent information (with respect to single-mode thermal states) of other Gaussian channels that are neither degradable nor anti-degradable, such as additive Gaussian noise channels and noisy amplification channels, also exhibit a nontrivial behavior similarly to the thermal-loss channels (see Methods for the definition of these other channels). More specifically, the coherent information of the additive Gaussian noise channel ${{\mathcal{N}}}_{{{\rm{B}}}_{2}}[\sigma ]$ is given by

11 $${I}_{{\rm{c}}}({{\mathcal{N}}}_{{{\rm{B}}}_{2}}[\sigma ],\hat{\tau }(\bar{n}))= 	\hskip 2pt g(\bar{n}+{\sigma }^{2})-g\left(\frac{D^{\prime} +{\sigma }^{2}-1}{2}\right)\\ 	-g\left(\frac{D^{\prime} -{\sigma }^{2}-1}{2}\right),$$

where $D^{\prime} \equiv \sqrt{{(2\bar{n}+{\sigma }^{2}+1)}^{2}-4\bar{n}(\bar{n}+1)}$. Also, the coherent information of the noisy amplifier channel ${\mathcal{A}}[G,{\bar{n}}_{{\rm{th}}}]$ is given by

12 $${I}_{{\rm{c}}}({\mathcal{A}}[G,{\bar{n}}_{{\rm{th}}}],\hat{\tau }(\bar{n}))= 	\hskip 2ptg(G\bar{n}+(G-1)({\bar{n}}_{{\rm{th}}}+1))\\ 	-g\left(\frac{D^{\prime\prime} +(G-1)(\bar{n}+{\bar{n}}_{{\rm{th}}}+1)-1}{2}\right)\\ 	-g\left(\frac{D^{\prime\prime} -(G-1)(\bar{n}+{\bar{n}}_{{\rm{th}}}+1)-1}{2}\right),$$

where

13 $$D^{\prime\prime} \equiv \sqrt{{((G+1)\bar{n}+(G-1)({\bar{n}}_{{\rm{th}}}+1)+1)}^{2}-4G\bar{n}(\bar{n}+1)}.$$

As can be seen from Fig. 3b, c, the coherent information of these other channels also exhibit the same convex behavior in the small $\bar{n}$ regime. Therefore, higher quantum state transmission rates can be achieved for these other Gaussian channels, as well by using the correlated multimode thermal states instead of using the single-mode thermal states, analogously to the case of thermal-loss channels as shown here.

### Reverse coherent information and two-way quantum capacity

We show that a similar technique can be used to establish an improved lower bound of the two-way quantum capacity of thermal-loss channels. Let ${I}_{{\rm{rc}}}({\mathcal{N}},\hat{\rho })$ be the reverse coherent information of a bosonic channel ${\mathcal{N}}$ with respect to an input state $\hat{\rho }$^45^.

14 $${I}_{{\rm{rc}}}({\mathcal{N}},\hat{\rho })\equiv S(\hat{\rho })-S\left({{\mathcal{N}}}^{{\rm{c}}}(\hat{\rho })\right).$$

Both the coherent information and the reverse coherent information of a channel ${\mathcal{N}}$ are lower bounds of the channel’s two-way quantum capacity:

15 $${C}_{{\rm{Q}},\leftrightarrow }({\mathcal{N}})\ge \mathop{{\mathrm{max}}}\limits_{\hat{\rho }}\left[{\mathrm{max}}\left({I}_{{\rm{c}}}({\mathcal{N}},\hat{\rho }),{I}_{{\rm{rc}}}({\mathcal{N}},\hat{\rho })\right)\right].$$

In the energy-constrained cases, the maximization should be performed over all input states that satisfy the energy constraint.

The best-known lower bound (before our work) of the two-way quantum capacity of a thermal-loss channel is either the channel’s coherent information or reverse coherent information with respect to a single-mode thermal state, i.e.,

16 $${C}_{{\rm{Q}},\leftrightarrow }^{\le \bar{n}}({\mathcal{N}}[\eta ,{\bar{n}}_{{\rm{th}}}])\ge \left\{\begin{array}{ll}{I}_{{\rm{c}}}({\mathcal{N}}[\eta ,{\bar{n}}_{{\rm{th}}}],\hat{\tau }(\bar{n}))&\bar{n}\le {\bar{n}}_{{\rm{th}}}\\ {I}_{{\rm{rc}}}({\mathcal{N}}[\eta ,{\bar{n}}_{{\rm{th}}}],\hat{\tau }(\bar{n}))&\bar{n} \, > \, {\bar{n}}_{{\rm{th}}}\end{array}\right.,$$

where ${I}_{{\rm{c}}}({\mathcal{N}}[\eta ,{\bar{n}}_{{\rm{th}}}],\hat{\tau }(\bar{n}))$ is given in Eq. (7) and ${I}_{{\rm{rc}}}({\mathcal{N}}[\eta ,{\bar{n}}_{{\rm{th}}}],\hat{\tau }(\bar{n}))$ can be obtained by replacing $g(\eta \bar{n}+(1-\eta ){\bar{n}}_{{\rm{th}}})$ in Eq. (7) by $g(\bar{n})$^29,45^, i.e.,

17 $${I}_{{\rm{rc}}}({\mathcal{N}}[\eta ,{\bar{n}}_{{\rm{th}}}],\hat{\tau }(\bar{n}))= \, g(\bar{n})-g\left(\frac{D+(1-\eta )(\bar{n}-{\bar{n}}_{{\rm{th}}})-1}{2}\right)\\ -\,g\left(\frac{D-(1-\eta )(\bar{n}-{\bar{n}}_{{\rm{th}}})-1}{2}\right).$$

In the special case where ${\bar{n}}_{{\rm{th}}}=0$ and $\bar{n}\to \infty$, the lower bound in Eq. (16) is given by  − log_2_(1 − *η*)^45^ and coincides with the upper bound established in ref. ^29^. Except for this special case, it is an open question whether the lower bound in Eq. (16) equals the true two-way quantum capacity of thermal-loss channels: here, we provide a negative answer to this question by showing that higher two-way quantum state transmission rate can be achieved by using correlated multimode thermal states.

**Theorem 2** *Consider a correlated N-mode thermal state* $\hat{{\mathcal{T}}}({\bf{N}},{\bf{n}})$ *with* **N** = (*M*, *N* − *M*) *and* ${\bf{n}}=({\bar{n}}_{1},{\bar{n}}_{2})$ *such that* $M{\bar{n}}_{1}+(N-M){\bar{n}}_{2}=N\bar{n}$ *and let* $x=\frac{M}{N}$, *where* *M* ∈ {1, ⋯ , *N*}. *The following two-way quantum state transmission rate can be achieved by using* $\hat{{\mathcal{T}}}({\bf{N}},{\bf{n}})$ *and hybridizing forward and backward strategies*:

18 $$\frac{M}{N}{I}_{{\rm{c}}}({\mathcal{N}}[\eta ,{\bar{n}}_{{\rm{th}}}],\hat{\tau }({\bar{n}}_{1}))+\frac{N-M}{N}{I}_{{\rm{rc}}}({\mathcal{N}}[\eta ,{\bar{n}}_{{\rm{th}}}],\hat{\tau }({\bar{n}}_{2})).$$

Since *x* can be any rational number in (0, 1] and the set of rational numbers is a dense subset of the set of real numbers, we have the following improved lower bound for the two-way energy-constrained quantum capacity of a thermal-loss channel:

19 $${C}_{{\rm{Q}},\leftrightarrow }^{\le \bar{n}}({\mathcal{N}}[\eta ,{\bar{n}}_{{\rm{th}}}])\ge 	\mathop{{\mathrm{max}}}\limits_{x,{\bar{n}}_{1},{\bar{n}}_{2}}\left[x{I}_{{\rm{c}}}({\mathcal{N}}[\eta ,{\bar{n}}_{{\rm{th}}}],\hat{\tau }({\bar{n}}_{1}))\right.\\ 	\left.+ \, (1-x){I}_{{\rm{rc}}}({\mathcal{N}}[\eta ,{\bar{n}}_{{\rm{th}}}],\hat{\tau }({\bar{n}}_{2}))\right],$$

where the maximization is performed subject to 0 ≤ *x* ≤ 1 and $x{\bar{n}}_{1}+(1-x){\bar{n}}_{2}=\bar{n}$.

The proof of Theorem 2 is given in the Methods section. Our new bound in Eq. (19) is at least as tight as the previous bound in Eq. (16) because the previous bound can be realized by plugging in *x* = 1 and ${\bar{n}}_{1}=\bar{n}$ or *x* = 0 and ${\bar{n}}_{2}=\bar{n}$. Moreover, we show that in the noisy channel (near-zero capacity) regime, correlated multimode thermal states outperform single-mode thermal states of the same energy and thus our bound is strictly tighter than the previous bound.

**Fig. 4:**
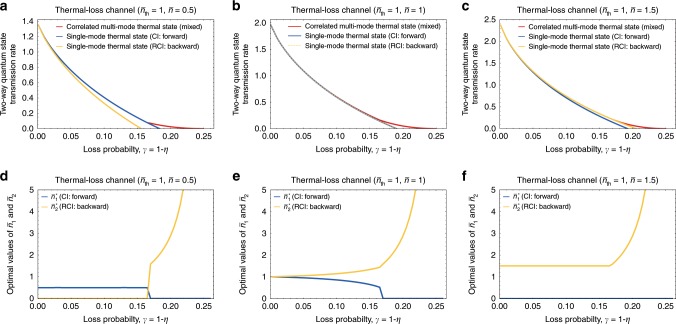
Achievable two-way quantum state transmission rate of thermal-loss channels. We plot the achievable two-way quantum state transmission rate of a thermal-loss channel ${\mathcal{N}}[\eta ,{\bar{n}}_{{\rm{th}}}=1]$ subject to the maximum allowed average photon number (**a**) $\bar{n}=0.5$ (**b**) $\bar{n}=1$ (**c**) $\bar{n}=1.5$ per channel use. In **a**–**c**, the blue and yellow lines, respectively, represent the coherent information ${I}_{{\rm{c}}}({\mathcal{N}}[\eta ,{\bar{n}}_{{\rm{th}}}],\hat{\tau }(\bar{n}))$ and the reverse coherent information ${I}_{{\rm{rc}}}({\mathcal{N}}[\eta ,{\bar{n}}_{{\rm{th}}}],\hat{\tau }(\bar{n}))$ with respect to single-mode thermal states. The achievable two-way quantum state transmission rate of correlated multimode thermal states (red lines in (**a**–**c**)) was evaluated by taking $({x}^{\star },{\bar{n}}_{1}^{\star },{\bar{n}}_{2}^{\star })={{\rm{argmax}}}_{x,{\bar{n}}_{1},{\bar{n}}_{2}}[x{I}_{{\rm{c}}}({\mathcal{N}}[\eta ,{\bar{n}}_{{\rm{th}}}],\hat{\tau }({\bar{n}}_{1}))+(1-x){I}_{{\rm{rc}}}({\mathcal{N}}[\eta ,{\bar{n}}_{{\rm{th}}}],\hat{\tau }({\bar{n}}_{2}))]$ subject to 0 ≤ *x* ≤ 1 and $x{\bar{n}}_{1}+(1-x){\bar{n}}_{2}=\bar{n}$ (see Theorem 2). In **d**–**f**, the optimal values ${\bar{n}}_{1}^{\star }$ and ${\bar{n}}_{2}^{\star }$ are, respectively, represented by the blue and yellow lines for (**d**) $\bar{n}=0.5$ (**e**) $\bar{n}=1$ (**f**) $\bar{n}=1.5$. The optimal value ${x}^{\star }$ can be obtained by evaluating ${x}^{\star }=(\bar{n}-{\bar{n}}_{2}^{\star })/({\bar{n}}_{1}^{\star }-{\bar{n}}_{2}^{\star })$.

To demonstrate that our new bound can be strictly tighter than the previous bound, we take a family of thermal-loss channels ${\mathcal{N}}[\eta ,{\bar{n}}_{{\rm{th}}}]$ with ${\bar{n}}_{{\rm{th}}}=1$. Then, we compute the new bound in Eq. (19) for each *η* = 1 − *γ* for three different maximum allowed average photon numbers per channel use, i.e., $\bar{n}=0.5$ (Fig. 4a), $\bar{n}=1$ (Fig. 4b), and $\bar{n}=1.5$ (Fig. 4c). As can be seen from the top panel of Fig. 4, the coherent information ${I}_{{\rm{rc}}}({\mathcal{N}}[\eta ,{\bar{n}}_{{\rm{th}}}],\hat{\tau }(\bar{n}))$ (blue lines) is larger than, equal to, and smaller than the reverse coherent information ${I}_{{\rm{rc}}}({\mathcal{N}}[\eta ,{\bar{n}}_{{\rm{th}}}],\hat{\tau }(\bar{n}))$ (yellow lines) for $\bar{n}=0.5$, $\bar{n}=1$, and $\bar{n}=1.5$, respectively. In all cases, our new bound obtained by using correlated multimode thermal states (red lines) can be strictly tighter than the previous bound in the large loss probability regime, where the two-way quantum capacity almost vanishes. In this regime, the best two-way quantum state transmission rate is achieved by mixing forward (coherent information) and backward (reverse coherent information) strategies, as can be seen from the bottom panels of Fig. 4.

### Private information and private capacity

Lastly, we apply our general technique to improve the lower bound of the energy-constrained private capacity of the thermal-loss channel. Consider a classical-quantum state $\hat{\sigma }=\int dxp(x)\left|x\right\rangle \left\langle x\right|\otimes {\hat{\rho }}_{x}$, where $\langle x| x^{\prime} \rangle =\delta (x-x^{\prime} )$ and ∫*dxp*(*x*) = 1 and let $\hat{\rho }\equiv \int dxp(x){\hat{\rho }}_{x}$. The private capacity of a quantum channel characterizes the channel’s maximum achievable secure classical communication rate. The private information of channel ${\mathcal{N}}$ with respect to the classical-quantum state $\hat{\sigma }$ is defined as

20 $${I}_{{\rm{p}}}({\mathcal{N}},\hat{\sigma })\equiv 	\hskip 2ptS\left({\mathcal{N}}(\hat{\rho })\right)-S\left({{\mathcal{N}}}^{{\rm{c}}}(\hat{\rho })\right)\\ 	-\int dxp(x)\left[S\left({\mathcal{N}}({\hat{\rho }}_{x})\right)-S\left({{\mathcal{N}}}^{{\rm{c}}}({\hat{\rho }}_{x})\right)\right].$$

The private capacity ${C}_{{\rm{P}}}({\mathcal{N}})$ of a quantum channel ${\mathcal{N}}$ is equal to the channel’s regularized private information ${P}_{{\rm{reg}}}({\mathcal{N}})$^13^:

21 $${C}_{{\rm{P}}}({\mathcal{N}})={P}_{{\rm{reg}}}({\mathcal{N}})\equiv \mathop{\mathrm{lim}}\limits_{N\to \infty }\frac{1}{N}\mathop{\mathrm{max}}\limits_{\hat{\sigma }}{I}_{{\rm{p}}}({{\mathcal{N}}}^{\otimes N},\hat{\sigma }).$$

In the energy-constrained case, the maximization should be performed over all classical-quantum states $\hat{\sigma }=\int dxp(x)\left|x\right\rangle \left\langle x\right|\otimes {\hat{\rho }}_{x}$ such that $\hat{\rho }=\int dxp(x){\hat{\rho }}_{x}$ satisfies the energy constraint.

The quantum capacity of a channel ${\mathcal{N}}$ is always a lower bound of the channel’s private capacity^13^ and thus the coherent information ${I}_{{\rm{c}}}({\mathcal{N}}[\eta ,{\bar{n}}_{{\rm{th}}}],\hat{\tau }(\bar{n}))$ in Eq. (7) is also a lower bound of the private capacity of the thermal-loss channel ${\mathcal{N}}[\eta ,{\bar{n}}_{{\rm{th}}}]$. Correspondingly, our new bound of the quantum capacity in Theorem 1 is also a valid lower bound of the private capacity which can be strictly tighter than ${I}_{{\rm{c}}}({\mathcal{N}}[\eta ,{\bar{n}}_{{\rm{th}}}],\hat{\tau }(\bar{n}))$. However, it was shown that higher secure classical communication rate (than the coherent information ${I}_{{\rm{c}}}({\mathcal{N}}[\eta ,{\bar{n}}_{{\rm{th}}}],\hat{\tau }(\bar{n}))$) can be achieved by using an ensemble of displaced thermal states^47^. More specifically, by using a classical-quantum state

22 $$\hat{\sigma }({\bar{n}}_{1},{\bar{n}}_{2})\equiv \int {d}^{2}\alpha \frac{{e}^{-| \alpha {| }^{2}/{\bar{n}}_{1}}}{\pi {\bar{n}}_{1}}\left|\alpha \right\rangle \left\langle \alpha \right|\otimes \hat{D}(\alpha )\hat{\tau }({\bar{n}}_{2}){\hat{D}}^{\dagger }(\alpha )$$

such that ${\bar{n}}_{1}+{\bar{n}}_{2}=\bar{n}$, where *α* = *α*_R_ + *iα*_I_ and $\langle \alpha | \alpha ^{\prime} \rangle ={\delta }^{(2)}(\alpha -\alpha ^{\prime} )$, the private communication rate

23 $${I}_{{\rm{p}}}({\mathcal{N}}[\eta ,{\bar{n}}_{{\rm{th}}}],\hat{\sigma })=	\, {I}_{{\rm{c}}}({\mathcal{N}}[\eta ,{\bar{n}}_{{\rm{th}}}],\hat{\tau }({\bar{n}}_{1}+{\bar{n}}_{2}))\\ 	-{I}_{{\rm{c}}}({\mathcal{N}}[\eta ,{\bar{n}}_{{\rm{th}}}],\hat{\tau }({\bar{n}}_{2}))$$

can be achieved. Thus, we have the following lower bound of the energy-constrained private capacity of thermal-loss channels:

24 $${C}_{{\rm{P}}}^{\le \bar{n}}({\mathcal{N}}[\eta ,{\bar{n}}_{{\rm{th}}}])\ge 	\hskip 2pt f(\eta ,{\bar{n}}_{{\rm{th}}},\bar{n})\\ \equiv	 \mathop{{\mathrm{max}}}\limits_{0\le {\bar{n}}_{2}\le \bar{n}}\left[{I}_{{\rm{c}}}({\mathcal{N}}[\eta ,{\bar{n}}_{{\rm{th}}}],\hat{\tau }(\bar{n}))\right.\\ 	\left. -{I}_{{\rm{c}}}({\mathcal{N}}[\eta ,{\bar{n}}_{{\rm{th}}}],\hat{\tau }({\bar{n}}_{2}))\right].$$

**Fig. 5:**
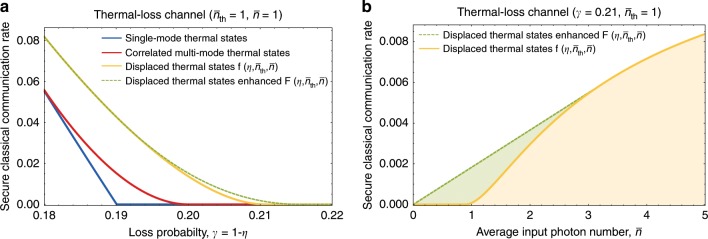
Achievable private communication rate of thermal-loss channels. **a** Achievable private communication rate of a thermal-loss channel ${\mathcal{N}}[\eta ,{\bar{n}}_{{\rm{th}}}]$ with ${\bar{n}}_{{\rm{th}}}=1$ subject to the maximum allowed average photon number $\bar{n}=1$ per channel use. The blue and red lines represent the achievable rates with the single-mode thermal state (Eq. (7)) and correlated multimode thermal states (Eq. (9)), respectively, and are identical to the blue and red lines in Fig. 2a. The yellow line represents the lower bound of ref. ^47^ ($f(\eta ,{\bar{n}}_{{\rm{th}}},\bar{n})$; see also Eq. (24)) obtained by using the displaced thermal state in Eq. (22). The dashed green line represents our improved lower bound ($F(\eta ,{\bar{n}}_{{\rm{th}}},\bar{n})$; see also Eq. (25)) obtained by using the classical-quantum state we constructed in Eq. (39). **b** Achievable private communication rate of a thermal-loss channel ${\mathcal{N}}[\eta ,{\bar{n}}_{{\rm{th}}}]$ with *η* =  0.79 (or *γ* = 0.21) and ${\bar{n}}_{{\rm{th}}}=1$ as a function of the maximum allowed average photon number $\bar{n}$. The yellow line represents the bound of ref. ^47^ ($f(\eta ,{\bar{n}}_{{\rm{th}}},\bar{n})$) and the dashed green line represents our bound ($F(\eta ,{\bar{n}}_{{\rm{th}}},\bar{n})$). Similarly as in the case of quantum state transmission (Figs. 2, 3), the nontrivial advantage of our classical-quantum state in Eq. (25) is due to the fact that $f(\eta ,{\bar{n}}_{{\rm{th}}},\bar{n})$ is convex in the small $\bar{n}$ regime, and thus the convex hull of the achievable region $\{(\bar{n},R)| \bar{n}\ \ge \ 0 \ {\rm{and}} \ R\ \le \ f(\eta ,{\bar{n}}_{{\rm{th}}},\bar{n})\}$ properly contains the region itself (see also the main text).

Since the coherent information ${I}_{{\rm{c}}}({\mathcal{N}}[\eta ,{\bar{n}}_{{\rm{th}}}],\hat{\tau }(\bar{n}))$ can be recovered by plugging in ${\bar{n}}_{2}=0$, this bound is at least as tight as the coherent information bound. In the noisy channel regime, the bound in Eq. (24) is strictly tighter than the coherent information bound (see the blue and yellow lines in Fig. 5a). Moreover, it is also strictly larger than our new bound for the quantum capacity in Eq. (9) (see the red and yellow lines in Fig. 5a). Therefore, our new bound for the quantum capacity is not the tightest lower bound for the private capacity. Nevertheless, we show below that our general technique can also be used to further improve the bound in Eq. (24). We establish the following result.

**Theorem 3** *The energy-constrained private capacity of a thermal-loss channel is lower bounded as follows*:

25 $${C}_{{\rm{P}}}^{\le \bar{n}}({\mathcal{N}}[\eta ,{\bar{n}}_{{\rm{th}}}])\ge F(\eta ,{\bar{n}}_{{\rm{th}}},\bar{n})\equiv \mathop{{\mathrm{max}}}\limits_{0<x\le 1}xf\left(\eta ,{\bar{n}}_{{\rm{th}}},\frac{\bar{n}}{x}\right),$$

where $f(\eta ,{\bar{n}}_{{\rm{th}}},\bar{n})$ is defined in Eq. (24).

The proof of Theorem 3 is given in the Methods section. As can be seen from Fig. 5a, our new bound in Eq. (25) is strictly tighter than the bound in Eq. (24) in the noisy channel regime. Similarly as above, this nontrivial advantage is due to the convexity of the function $f(\eta ,{\bar{n}}_{{\rm{th}}},\bar{n})$ in $\bar{n}$ in the small $\bar{n}$ regime for fixed values of *η* and ${\bar{n}}_{{\rm{th}}}$. For illustration, we plot in Fig. 5b the function $f(\eta ,{\bar{n}}_{{\rm{th}}},\bar{n})$ as a function of $\bar{n}$ for *η* = 0.79 (or *γ* = 0.21) and ${\bar{n}}_{{\rm{th}}}=1$. Due to the convexity of $f(\eta ,{\bar{n}}_{{\rm{th}}},\bar{n})$ in the small $\bar{n}$ regime, the convex hull of the achievable region $\{(\bar{n},R)| \bar{n}\ \ge \ 0\,{\rm{and}}\,R\ \le \ f(\eta ,{\bar{n}}_{{\rm{th}}},\bar{n})\}$ properly contains the region itself (see the shaded green region in Fig. 5b) and this explains the superior performance of our new bound in Eq. (25).

## Discussion

In this work, we have improved the lower bounds to the various energy-constrained quantum capacities of the thermal-loss channel, which is the basic model for realistic optical and microwave communication channels. In this way, our work shows that higher communication rates can be achieved for various quantum communication tasks with these practically relevant quantum channels than previously believed. Below, we make several remarks and discuss possible new research directions.

First, it was shown in a related work^53^ that a global encoding scheme with a correlated Gaussian input state can yield larger coherent information than a local encoding scheme with an uncorrelated Gaussian input state for lossy bosonic channels with correlated environmental noise. We remark that our work differs from this previous work in that we show a correlated Gaussian input state can outperform its uncorrelated counterpart even for the usual thermal-loss channels with uncorrelated environmental noise. Note that the loss model with uncorrelated environmental noise which we consider here has greater practical relevance because noise in realistic optical and microwave communication channels is well approximated by thermal-loss channels with uncorrelated environmental thermal noise^40,41^.

In addition, our result in Theorem 1 can be understood as the establishment of the superadditivity of the coherent information of thermal-loss channels with respect to Gaussian input states: as shown in ref. ^38^, the single-mode thermal state $\hat{\tau }(\bar{n})$ is the optimal single-mode Gaussian input state for the coherent information of thermal-loss channels. Since we show that multimode correlated thermal states (which are Gaussian) sometimes outperform the single-mode thermal state, it means that the coherent information of thermal-loss channels is superadditive with respect to Gaussian input states. On the other hand, it is still unclear whether the coherent information of thermal-loss channels is genuinely superadditive with respect to all input states. This is because technically there is still a possibility that some non-Gaussian input state may outperform all Gaussian input states. We leave this optimality question in the non-Gaussian domain as an open research direction.

Another interesting open question is whether the convexity argument presented here can be adapted to explain the known superadditivity behavior of the qubit depolarization^18,54,55^ and dephrasure^56–58^ channels. To contrast, we remark that the coherent information of a degradable channel is concave with respect to input states and its quantum capacity is additive^26–28^ (see also ref. ^59^).

We also remark that our improvement of the lower bounds is not strong enough to close the gap between the lower bounds and the best-known upper bounds of various energy-constrained quantum capacities of thermal-loss channels^29,47–49^. It will thus be interesting to see whether it is possible to further improve the lower and upper bounds to get a better understanding of the various quantum capacities of thermal-loss channels.

Finally, we emphasize that we did not provide explicit strategies to achieve various quantum communication rates established in this work, but only proved their existence. This is because the achievability of the coherent information, reverse coherent information, and private information is based on random coding arguments. Therefore, it will be an interesting research avenue to look for explicit quantum communication protocols (e.g., by using GKP codes^60,61^ or polar codes^62,63^) that can be implemented efficiently while achieving (or even improving) the rates we have established here.

## Methods

### Gaussian states and channels

A bosonic mode is described by its annihilation and creation operators $\hat{a}$ and ${\hat{a}}^{\dagger }$ that satisfy the commutation relation $[\hat{a},{\hat{a}}^{\dagger }]=1$ (see, for example, ref. ^35^). Let $\hat{{\bf{x}}}\equiv ({\hat{q}}_{1},\cdots \ ,{\hat{q}}_{N},{\hat{p}}_{1},\cdots \ ,{\hat{p}}_{N})$ be the quadrature operators of *N* bosonic modes, where ${\hat{q}}_{k}\equiv ({\hat{a}}_{k}^{\dagger }+{\hat{a}}_{k})/\sqrt{2}$ and ${\hat{p}}_{k}\equiv i({\hat{a}}_{k}^{\dagger }-{\hat{a}}_{k})/\sqrt{2}$. The quadrature operators satisfy the commutation relation $[{\hat{{\bf{x}}}}_{j},{\hat{{\bf{x}}}}_{k}]=i{\Omega }_{jk}$, where Ω is defined as

26 $$\Omega =\left[\begin{array}{ll}0&{I}_{N}\\ -{I}_{N}&0\end{array}\right]$$

and *I*_*N*_ is the *N* × *N* identity matrix.

By definition, the characteristic function of a Gaussian state $\hat{\rho }$ is Gaussian^36^:

27 $${\chi }_{\hat{\rho }}({\boldsymbol{\xi }})\equiv 	\hskip 2pt {\rm{Tr}}\left[\right.\hat{\rho }{\mathrm{exp}}[i{\hat{{\bf{x}}}}^{T}\Omega {\boldsymbol{\xi }}]\left]\right.\\ =	{\mathrm{exp}}\left[-\frac{1}{2}{{\boldsymbol{\xi }}}^{T}(\Omega V{\Omega }^{T}){\boldsymbol{\xi }}-i{(\Omega \bar{{\bf{x}}})}^{T}{\boldsymbol{\xi }}\right],$$

where $\bar{{\bf{x}}}$ and *V* are the first and the second moments of the quadrature operator $\hat{{\bf{x}}}$. A Gaussian state is thus fully characterized by its first two moments and one can write $\hat{\rho }={\hat{\rho }}_{{\rm{G}}}(\bar{{\bf{x}}},V)$.

A Gaussian channel ${\mathcal{N}}$ is a completely positive and trace preserving map (a CPTP map)^64^ that maps a Gaussian state ${\hat{\rho }}_{{\rm{G}}}(\bar{{\bf{x}}},V)$ to another Gaussian state ${\hat{\rho }}_{{\rm{G}}}(\bar{{\bf{x}}}^{\prime} ,V^{\prime} )$. A Gaussian channel ${\mathcal{N}}$ is fully characterized by its action on Gaussian states,

28 $$\bar{{\bf{x}}}^{\prime} =	T\bar{{\bf{x}}}+{\bf{d}},\\ V^{\prime}=	 TV{T}^{T}+\bar{N},$$

i.e., by $(T,\bar{N},{\bf{d}})$. A thermal-loss channel ${\mathcal{N}}[\eta ,{\bar{n}}_{{\rm{th}}}]$ is a single-mode Gaussian channel that has $T=\sqrt{\eta }{I}_{2}$, $\bar{N}=(1-\eta )({\bar{n}}_{{\rm{th}}}+\frac{1}{2}){I}_{2}$, and **d** = **0**, where *η* ∈ [0, 1] and ${\bar{n}}_{{\rm{th}}}\ \ge \ 0$. Thermal-loss channels are a good model for realistic optical and microwave communication channels. A thermal-loss channel with ${\bar{n}}_{{\rm{th}}}=0$ is called a bosonic pure-loss channel.

Other single-mode Gaussian channels include additive Gaussian noise channels and amplifier channels. An additive Gaussian noise channel ${{\mathcal{N}}}_{{{\rm{B}}}_{2}}[\sigma ]$ is characterized by *T* = *I*_2_, $\bar{N}={\sigma }^{2}{I}_{2}$, and **d** = **0**, and is also called a Gaussian random displacement channel. An amplifier channel ${\mathcal{A}}[G,{\bar{n}}_{{\rm{th}}}]$ is characterized by $T=\sqrt{G}{I}_{2}$, $\bar{N}=(G-1)({\bar{n}}_{{\rm{th}}}+\frac{1}{2}){I}_{2}$, and **d** = **0** where *G* ≥ 1. An amplifier channel is called a quantum-limited amplification channel, if ${\bar{n}}_{{\rm{th}}}=0$ and a noisy amplification channel if ${\bar{n}}_{{\rm{th}}}\ > \ 0$ (see Sec. V of ref. ^36^ for more details).

### Gaussian Fourier transformation

We define the *N*-mode Gaussian Fourier transformation ${\hat{U}}_{{\rm{GFT}}}^{(N)}$ as a Gaussian operation that transforms the annihilation operators by a discrete Fourier transformation:

29 $${\left({\hat{U}}_{{\rm{GFT}}}^{(N)}\right)}^{\dagger }{\hat{a}}_{j}{\hat{U}}_{{\rm{GFT}}}^{(N)}=\frac{1}{\sqrt{N}}\sum _{k=1}^{N}{e}^{i\frac{2\pi }{N}(j-1)(k-1)}{\hat{a}}_{k}.$$

The *N*-mode Gaussian Fourier transformation can also be understood as a Gaussian (unitary) channel that is characterized by

30 $$T=\left[\begin{array}{llll}R(0)&R(0)&\cdots \ &R(0)\\ R(0)&R(\frac{2\pi }{N})&\cdots \ &R(\frac{2\pi }{N}(N-1))\\ \vdots &\vdots &\ddots &\vdots \\ R(0)&R(\frac{2\pi }{N}(N-1))&\cdots \ &R(\frac{2\pi }{N}{(N-1)}^{2})\end{array}\right],$$

*N* = 0, and **d** = 0, where

31 $$R(\theta )\equiv \left[\begin{array}{ll}{\mathrm{cos}} \, \theta &-{\mathrm{sin}} \, \theta \\ {\mathrm{sin}} \, \theta &{\mathrm{cos}} \, \theta \end{array}\right].$$

Since *T* is an orthogonal matrix (i.e., *TT*^*T*^ = *T*^*T*^*T* = *I*_2*N*_), the *N*-mode Gaussian Fourier transformation is a passive linear optical operation that does not require squeezing.

### Proofs of Theorems 1 and 2

Let ${{\mathcal{U}}}_{{\rm{GFT}}}^{(N)}(\hat{\rho })\equiv {\hat{U}}_{{\rm{GFT}}}^{(N)}\hat{\rho }{({\hat{U}}_{{\rm{GFT}}}^{(N)})}^{\dagger }$ be the unitary quantum channel associated with the *N*-mode Gaussian Fourier transformation. Then, ${{\mathcal{U}}}_{{\rm{GFT}}}^{(N)}$ commutes with the tensor product of thermal-loss channels, i.e.,

32 $${{\mathcal{U}}}_{{\rm{GFT}}}^{(N)}{\mathcal{N}}{[\eta ,{\bar{n}}_{{\rm{th}}}]}^{\otimes N}={\mathcal{N}}{[\eta ,{\bar{n}}_{{\rm{th}}}]}^{\otimes N}{{\mathcal{U}}}_{{\rm{GFT}}}^{(N)}.$$

This is a direct consequence of the fact that the *N*-mode Gaussian Fourier transformation is a passive linear optical operation with an orthogonal transformation matrix *T*. Now, recall that the correlated multimode thermal state $\hat{{\mathcal{T}}}({\bf{N}},{\bf{n}})$ with **N** = (*N*_1_, ⋯ , *N*_*r*_) and ${\bf{n}}=({\bar{n}}_{1},\cdots \ ,{\bar{n}}_{r})$ is defined as

33 $$\hat{{\mathcal{T}}}({\bf{N}},{\bf{n}})={{\mathcal{U}}}_{{\rm{GFT}}}^{(N)}\left({\left\{\hat{\tau }({\bar{n}}_{1})\right\}}^{\otimes {N}_{1}}\otimes \cdots \otimes {\left\{\hat{\tau }({\bar{n}}_{r})\right\}}^{\otimes {N}_{r}}\right).$$

Combining Eq. (32) and Eq. (33), one can see that sending the correlated multimode thermal state $\hat{{\mathcal{T}}}({\bf{N}},{\bf{n}})$ to the *N* copies of thermal-loss channels is equivalent to sending a collection of thermal states ${\left\{\hat{\tau }({\bar{n}}_{1})\right\}}^{\otimes {N}_{1}}\otimes \cdots \otimes {\left\{\hat{\tau }({\bar{n}}_{r})\right\}}^{\otimes {N}_{r}}$ to the thermal-loss channels and then the receiver performing the Gaussian Fourier transformation. Since any local operations are assumed to be free, the achievable communication rates with the correlated multimode thermal state $\hat{{\mathcal{T}}}({\bf{N}},{\bf{n}})$ is the same as the rates achievable with the collection of thermal states ${\left\{\hat{\tau }({\bar{n}}_{1})\right\}}^{\otimes {N}_{1}}\otimes \cdots \otimes {\left\{\hat{\tau }({\bar{n}}_{r})\right\}}^{\otimes {N}_{r}}$.

For quantum state transmission without any classical feedback assistance, the achievable rate is given by the coherent information. Since

34 $${I}_{{\rm{c}}}({\mathcal{N}}{[\eta ,{\bar{n}}_{{\rm{th}}}]}^{\otimes N},\hat{{\mathcal{T}}}({\bf{N}},{\bf{n}}))=	\,{I}_{{\rm{c}}}\left({\mathcal{N}}{[\eta ,{\bar{n}}_{{\rm{th}}}]}^{\otimes N},{\left\{\hat{\tau }({\bar{n}}_{1})\right\}}^{\otimes {N}_{1}}\otimes \cdots \otimes {\left\{\hat{\tau }({\bar{n}}_{r})\right\}}^{\otimes {N}_{r}}\right)\\ =	\sum _{k=1}^{r}{N}_{k}{I}_{{\rm{c}}}({\mathcal{N}}[\eta ,{\bar{n}}_{{\rm{th}}}],\hat{\tau }({\bar{n}}_{k})),$$

the correlated multimode thermal state $\hat{{\mathcal{T}}}({\bf{N}},{\bf{n}})$ can achieve the quantum state transmission rate

35 $$\frac{1}{N}\sum _{k=1}^{r}{N}_{k}{I}_{{\rm{c}}}({\mathcal{N}}[\eta ,{\bar{n}}_{{\rm{th}}}],\hat{\tau }({\bar{n}}_{k}))$$

per channel use. Specializing this to **N** = (*M*, *N* − *M*) and ${\bf{n}}=(\frac{N}{M}\bar{n},0)$, we get the rate

36 $$\frac{M}{N}{I}_{{\rm{c}}}\left({\mathcal{N}}[\eta ,{\bar{n}}_{{\rm{th}}}],\hat{\tau }\left(\frac{N}{M}\bar{n}\right)\right)=x{I}_{{\rm{c}}}\left({\mathcal{N}}[\eta ,{\bar{n}}_{{\rm{th}}}],\hat{\tau }\left(\frac{\bar{n}}{x}\right)\right)$$

as stated in Eq. (8) in Theorem 1, where *x* ≡ *M*∕*N*. Following the rest of the arguments given in Theorem 1, the theorem follows.

Note that it might appear that the use of Gaussian Fourier transformation is not necessary because as shown in Eq. (34), the coherent information of the correlated multimode thermal state $\hat{{\mathcal{T}}}({\bf{N}},{\bf{n}})$ is the same as the coherent information of the uncorrelated multimode thermal state ${\left\{\hat{\tau }({\bar{n}}_{1})\right\}}^{\otimes {N}_{1}}\otimes \cdots \otimes {\left\{\hat{\tau }({\bar{n}}_{r})\right\}}^{\otimes {N}_{r}}$. It is nevertheless essential to use the Gaussian Fourier transformation because it uniformly spreads the excessive photons in uncorrelated multimode thermal state across all modes such that the energy constraint is fulfilled (see also the discussion below Eq. (2)).

Now consider the two-way quantum state transmission rate and the correlated multimode thermal states given in Theorem 2, i.e., $\hat{{\mathcal{T}}}({\bf{N}},{\bf{n}})$ with **N** = (*M*, *N* − *M*) and ${\bf{n}}=({\bar{n}}_{1},{\bar{n}}_{2})$. As shown above, sending this state to a thermal-loss channel is equivalent to sending the collection of thermal states ${\left\{\hat{\tau }({\bar{n}}_{1})\right\}}^{\otimes M}\otimes {\left\{\hat{\tau }({\bar{n}}_{2})\right\}}^{\otimes N-M}$. Since classical feedback assistance is allowed, the reverse coherent information is also achievable in this case. By employing the forward strategy for the first *M* modes and the backward strategy for the last *N*−*M* modes, we can achieve the two-way quantum state transmission rate

37 $$\frac{M}{N}{I}_{{\rm{c}}}({\mathcal{N}}[\eta ,{\bar{n}}_{{\rm{th}}}],\hat{\tau }({\bar{n}}_{1}))+\frac{N-M}{N}{I}_{{\rm{rc}}}({\mathcal{N}}[\eta ,{\bar{n}}_{{\rm{th}}}],\hat{\tau }({\bar{n}}_{2}))$$

per channel use as stated in Eq. (18) in Theorem 2. Again, following the rest of the arguments in Theorem 2, the theorem follows.

### Proof of Theorem 3

Recall that the classical-quantum state

38 $$\hat{\sigma }({\bar{n}}_{1},{\bar{n}}_{2})\equiv \int {d}^{2}\alpha \frac{{e}^{-| \alpha {| }^{2}/{\bar{n}}_{1}}}{\pi {\bar{n}}_{1}}\left|\alpha \right\rangle \left\langle \alpha \right|\otimes \hat{D}(\alpha )\hat{\tau }({\bar{n}}_{2}){\hat{D}}^{\dagger }(\alpha )$$

with ${\bar{n}}_{1}+{\bar{n}}_{2}=\bar{n}$ was used to establish the recent lower bound in Eq. (24). Similar to construction of correlated multimode thermal states in Theorem 1, we construct the following classical-quantum state

39 $$\hat{\Sigma }\equiv {\left\{\hat{\sigma }\left(\frac{N}{M}\bar{n}-{\bar{n}}_{2}^{\star },{\bar{n}}_{2}^{\star }\right)\right\}}^{\otimes M}\otimes {\left\{\hat{\sigma }(0,0)\right\}}^{\otimes N-M},$$

where *M* ∈ {1, ⋯ , *N*}. Then, by choosing

40 $${\bar{n}}_{2}^{\star }= {\mathop{{\rm{argmin}}} _{0\le {\bar{n}}_{2}\le \frac{N}{M}\bar{n}}} \,{I}_{{\rm{c}}}({\mathcal{N}}[\eta ,{\bar{n}}_{{\rm{th}}}],{\bar{n}}_{2}),$$

we can see that the achievable private communication rate of the state $\hat{\Sigma }$ is given by

41 $$\frac{M}{N}f\left(\eta ,{\bar{n}}_{{\rm{th}}},\frac{N}{M}\bar{n}\right)=xf\left(\eta ,{\bar{n}}_{{\rm{th}}},\frac{\bar{n}}{x}\right)$$

per channel use where *x* = *M*∕*N* ∈ (0, 1] is a rational number. Since the set of rational numbers is a dense subset of the set of real numbers, the rate in Eq. (41) is achievable for any real number *x* ∈ (0, 1] and the theorem follows.

## Supplementary information

Peer Review File

## Data Availability

No data sets were generated during this study.

## Code availability

Source codes of the plots are available from the corresponding author upon request.
