# Publisher Correction: Enhanced energy-constrained quantum communication over bosonic Gaussian channels

**DOI:** 10.1038/s41467-020-15176-1

**Published:** 2020-03-11

**Authors:** Kyungjoo Noh, Stefano Pirandola, Liang Jiang

**Affiliations:** 1 Departments of Applied Physics and Physics, Yale University, New Haven, CT 06511 USA; 2 Yale Quantum Institute, Yale University, New Haven, CT 06520 USA; 3 Computer Science and York Centre for Quantum Technologies, University of York, York, YO10 5GH UK; 4 Research Laboratory of Electronics, Massachusetts Institute of Technology (MIT), Cambridge, MA 02139 USA; 5 Pritzker School of Molecular Engineering, University of Chicago, 5640 South Ellis Avenue, Chicago, IL 60637 USA

Correction to: *Nature Communications* 10.1038/s41467-020-14329-6, published online 23 January 2020.

The original pdf version of this Article contained an error in Eq. (9) and in Eq. (25), where the domain of “max” function was incorrectly given as “0 ≤ 1” instead of “0 < x ≤ 1” and in Caption of Fig. 2, where the domain of “argmax” function was also given as “0 ≤ 1” instead of “0 < x ≤ 1”. The pdf has been updated to include these corrections. The original HTML version was correct and has not been changed.

