# Supplementary material for: Enhanced energy-constrained quantum communication over bosonic Gaussian channels
Source: Nat Commun. 2020 Jan 23;11:457. doi: 10.1038/s41467-020-14329-6 (PMC6978311; doi:10.1038/s41467-020-14329-6)
Supplement: Supplementary file 1 — Peer Review File [file 41467_2020_14329_MOESM1_ESM.pdf]

Reviewers' Comments:

Reviewer #1:
Remarks to the Author:
The authors investigate achievable rates (under energy constraint) for communication over bosonic Gaussian channels, in particular thermal-loss channels. They find that multi-mode input states offer an advantage over single-mode states in the low energy regime. They thus improve the best-known lower bounds on the energy-constrained capacity of such channels. The constructions they use to show these bounds are elementary and the results are not entirely surprising, but certainly of interest to a specialised audience working on Gaussian quantum channels. One thing I find lacking is any comparison with upper bounds for the capacity. I am thus assuming that the achieved bounds are still far from the best known upper bounds. The paper is very well presented, clear and concise, and definitively publishable in its current form. I am however somewhat sceptical that it is suitable for Nature Communications.

Reviewer #2:
Remarks to the Author:
In the submission NCOMMS-19-6658204 with the title "Enhanced energy-constrained quantum communication over bosonic Gaussian channels", the authors prove new lower bounds on energy-constrained capacities of thermal loss channels. These bosonic channels characterize energy loss and thermal noise errors in optical and microwave communication links, and the energy constraint represents a realistic scenario in which the average number of photons per channel use is limited. The improved bounds on the capacities are achieved by using correlated multi-mode thermal states at the input. As the main technical contribution, the authors compute the regularized coherent information of this code family and show improvement on existing single-letter bounds on the one-way quantum capacity of the thermal loss channel. A similar technique is furthermore used to prove new lower bounds for the two-way capacity and private capacity, respectively, again improving on existing bounds in the literature. The authors also supply a convexity argument that sheds further light on the origin of the improvement achieved by correlated multi-mode thermal states.

To the best of my knowledge, all proofs given in the manuscript are correct. The paper is very well written and clearly develops the crucial points leading to the main results. The techniques used in this paper will most likely have further applications in bosonic/Gaussian quantum information, and the authors raise some interesting questions concerning connections to the discrete variable setting, in particular with respect to the convexity argument explaining the power of the new code family. This manuscript will be of interest for the wider quantum information theory community, in particular for people interested in continuous-variable quantum information processing. I therefore think that this paper is suitable for publication in Nature Communications.

Below is a list of some typos and minor suggestions for improvement of the manuscript.

* p.2, right col, third par: "In this paper, we establish improved lower bounds on various energy-constrained quantum capacities of thermal-loss channels *that are stronger* than the existing bounds.

* p.3, right col, second par: In the definition of the energy-constrained quantum capacity, putting the energy constraint on \hat{\rho} in the \max argument would make the equation a bit clearer in my opinion.

* p.4, right col, after eq.(10): the \lambda_k have to be \geq 0, not \leq 0. Moreover, are the \lambda_k in this step really arbitrary real non-negative numbers? Because it seems like the vector \vec{N} = N(\lambda_1, ..., \lambda_k) needs to be a vector of integers (but then a

density argument shows what the authors claim).

* p.5, right col, last par: "In *all* cases, ..."

* p.6, right col, first par: Remove the first 'also' in the sentence "Nevertheless, ..."

* p.6, right col, last par: The authors talk of 'superadditivity of thermal-loss channels' here, but I think it would be good to be more specific and say 'superadditivity of coherent information/private information/...'.

* p.7, left col, second par: I find the wording in the sentence "To contrast, we remark that the coherent information of a degradable channel is concave with respect to input states, so that its quantum capacity is additive." confusing, because it suggests that additive coherent information (which it should say here instead of quantum capacity) is a consequence of the concavity of coherent information. To the best of my knowledge this is not true, and both properties (concavity in input state and additivity of coherent information) individually follow from degradability of the quantum channel (proved in Ref.'s [26] and [28], respectively). In connection to this point, see also the results of Watanabe [Phys. Rev. A 85, 012326 (2012)].

* p.7, left col, third par: '*reverse* coherent information'

* p.7, right col, eq.(28): second \mathbf{T} should be transposed.

* p.7, right col, after eq.(28): The definition of \mathbf{N} for the thermal loss channel is missing a factor (1-\eta).

* p.8, left col, eq.(34): At first sight it seems as if the correlation induced by the GFT is not even needed in the proof, as the correlated multi-mode thermal states have the same coherent information as the (uncorrelated) collection of thermal states. But the GFT is indeed needed in order to spread the photon numbers across the modes and thus satisfy the energy constraint imposed on the quantum codes. I think it would be helpful for the reader to stress this once more at this point (the authors do point this out in the paragraph after eq.(2)).

* p.8, left col, third par: "same as the rates achievable with the collection *of* thermal states ..."

* p.8, right col, last par: "Since the set of rational numbers is *a* dense subset of the set of real numbers, ..."

* p.14, caption of Fig. 5: "Achievable private communcation rate of *a* thermal-loss channel"

(Referee report written by Felix Leditzky)

Reviewer #3:
Remarks to the Author:
The authors consider quantum and classical private communication via bosonic Gaussian channels under energy constraints. Their main results are improved lower bounds on various capacities for general thermal loss channels. In contrast to pure loss channels, which are either degradable or anti-degradable, such channels can have non-additive coherent information, hence their quantum capacities are non-trivial.

In this work the authors use a correlated multi-mode thermal state, instead of a tensor product of single-mode thermal states, as inputs and show that this can yield a higher coherent information, hence a better bound on the energy constraint quantum capacity. Combining this with a new

scheme of classical communication assistance, that combines forward and backward classical communication, they also obtain better lower bounds for the two-way classical communication assisted energy constraints quantum capacity. Finally, by constructing a classical-quantum-state, they provide a lower bound on the energy constraint private capacity that can outperform previous bounds.

As the authors point out in the discussion, it has already been shown in reference [52] that global encoding with correlated Gaussian states can result in a higher coherent information than product encoding. The significance of the present result is that it considers thermal loss channels with uncorrelated environmental noise, which are of greater practical relevance.

I believe the paper is interesting and well written, so it could be published. However the authors should explain in more detail how significant the difference is to the results presented in [52]. It might be clear to all the readers, why channels with uncorrelated environmental noise are of significantly greater relevance than correlated ones.

Response to the referees' comments

Kyungjoo Noh, Stefano Pirandola, and Liang Jiang

December 13, 2019

Reviewer #1 (Remarks to the Author):

The authors investigate achievable rates (under energy constraint) for communication over bosonic Gaussian channels, in particular thermal-loss channels. They find that multi-mode input states offer an advantage over single-mode states in the low energy regime. They thus improve the best-known lower bounds on the energy-constrained capacity of such channels. The constructions they use to show these bounds are elementary and the results are not entirely surprising, but certainly of interest to a specialised audience working on Gaussian quantum channels. One thing I find lacking is any comparison with upper bounds for the capacity. I am thus assuming that the achieved bounds are still far from the best known upper bounds. The paper is very well presented, clear and concise, and definitively publishable in its current form. I am however somewhat sceptical that it is suitable for Nature Communications.

Reply–We thank the reviewer for the careful reading of our manuscript and pointing out the missing comparison of our lower bounds with the existing upper bounds. it is indeed true that our improvement of the lower bounds is not strong enough to close the gap between the lower and upper bounds. In the revised manuscript, we added a paragraph about this in the discussion section (page 7, left column). Regarding the gap, however, we remark that the gap is due to the limitations of both the lower and the upper bounds. In particular, we would like to point out that the lower bound of the quantum capacity of thermal-loss channels has not been improved for a long time since the early work by Holevo and Werner in 2001 [Phys. Rev. A 63, 032312 (2001)]. On the other hand, we managed to go beyond these early results for the first time in about two decades. We would also like to stress that the we have provided a general explanation of our new results by using simple mathematical concepts such as convexity and convex hull. The convexity argument might shed further light on the superadditive behaviors of the quantum capacities of a broader class of quantum channels including discrete-variable quantum channels.

Reviewer #2 (Remarks to the Author):

In the submission NCOMMS-19-6658204 with the title "Enhanced energy-constrained quantum communication over bosonic Gaussian channels", the authors prove new lower bounds on energy-constrained capacities of thermal loss channels. These bosonic channels characterize energy loss and thermal noise errors in optical and microwave communication links, and the energy constraint represents a realistic scenario in which the average number of photons per channel use is limited. The improved bounds on the capacities are achieved by using correlated multi-mode thermal states at the input. As the main technical contribution, the authors compute the regularized coherent information of this code family and show improvement on existing single-letter bounds on the one-way quantum capacity of the thermal loss channel. A similar technique is furthermore used to prove new lower bounds for the two-way capacity and private capacity, respectively, again improving on existing bounds in the literature. The authors also supply a convexity argument that sheds further light on the origin of the improvement achieved by correlated multi-mode thermal states.

To the best of my knowledge, all proofs given in the manuscript are correct. The paper is very well written and clearly develops the crucial points leading to the main results. The techniques used in this paper will most likely have further applications in bosonic/Gaussian quantum information, and the authors raise some interesting questions concerning connections to the discrete variable setting, in particular with respect to the convexity argument explaining the power of the new code family. This manuscript will

be of interest for the wider quantum information theory community, in particular for people interested in continuous-variable quantum information processing. I therefore think that this paper is suitable for publication in Nature Communications.

Reply–We thank the reviewer for the appreciation of our work.

Below is a list of some typos and minor suggestions for improvement of the manuscript.

*p.2, right col, third par: "In this paper, we establish improved lower bounds on various energy-constrained quantum capacities of thermal-loss channels *that are stronger* than the existing bounds.*

p.3, right col, second par: In the definition of the energy-constrained quantum capacity, putting the energy constraint on $\hat{\rho}$ in the max *argument would make the equation a bit clearer in my opinion.*

Reply–We implemented these suggested changes in the revised manuscript.

p.4, right col, after eq.(10): the λ_k have to be ≥ 0, not ≤ 0. Moreover, are the λ_k in this step really arbitrary real non-negative numbers? Because it seems like the vector $\vec{N} = N(\lambda_1, ..., \lambda_k)$ needs to be a vector of integers (but then a density argument shows what the authors claim).

Reply–We corrected the type and revised the text below Eq. (10) to clarify why λ_k has to be rational numbers and how one can deal with irrational λ_k.

*p.5, right col, last par: "In *all* cases, ..."*

p.6, right col, first par: Remove the first 'also' in the sentence "Nevertheless, ..."

p.6, right col, last par: The authors talk of 'superadditivity of thermal-loss channels' here, but I think it would be good to be more specific and say 'superadditivity of coherent information/private information/...'.

Reply–We implemented these suggested changes in the revised manuscript.

p.7, left col, second par: I find the wording in the sentence "To contrast, we remark that the coherent information of a degradable channel is concave with respect to input states, so that its quantum capacity is additive." confusing, because it suggests that additive coherent information (which it should say here instead of quantum capacity) is a consequence of the concavity of coherent information. To the best of my knowledge this is not true, and both properties (concavity in input state and additivity of coherent information) individually follow from degradability of the quantum channel (proved in Ref.'s [26] and [28], respectively). In connection to this point, see also the results of Watanabe [Phys. Rev. A 85, 012326 (2012)].

Reply–We thank the reviewer for pointing this out. In the revised manuscript, we re-wrote the sentence to avoid the confusion and added a reference to [Phys. Rev. A 85, 012326 (2012)].

*p.7, left col, third par: '*reverse* coherent information'*

p.7, right col, eq.(28): second $\mathbf{T}$ *should be transposed.*

p.7, right col, after eq.(28): The definition of $\mathbf{N}$ *for the thermal loss channel is missing a factor* $(1-\eta)$.

Reply–We corrected these typos.

p.8, left col, eq.(34): At first sight it seems as if the correlation induced by the GFT is not even needed in the proof, as the correlated multi-mode thermal states have the same coherent information as the (uncorrelated) collection of thermal states. But the GFT is indeed needed in order to spread the photon numbers across the modes and thus satisfy the energy constraint imposed on the quantum codes. I think it would be helpful for the reader to stress this once more at this point (the authors do point this out in the paragraph after eq.(2)).

Reply–We agree that it would be good to stress this point once more in the method section so added a paragraph about this (page 8, right column). We thank the reviewer for bringing this to our attention.

*p.8, left col, third par: "same as the rates achievable with the collection *of* thermal states ..."*

*p.8, right col, last par: "Since the set of rational numbers is *a* dense subset of the set of real numbers, ..."*

*p.14, caption of Fig. 5: "Achievable private communcation rate of *a* thermal-loss channel"*

Reply–We corrected these typos.

(Referee report written by Felix Leditzky)

Reviewer #3 (Remarks to the Author):

The authors consider quantum and classical private communication via bosonic Gaussian channels under energy constraints. Their main results are improved lower bounds on various capacities for general thermal loss channels. In contrast to pure loss channels, which are either degradable or anti-degradable, such channels can have non-additive coherent information, hence their quantum capacities are non-trivial.

In this work the authors use a correlated multi-mode thermal state, instead of a tensor product of single-mode thermal states, as inputs and show that this can yield a higher coherent information, hence a better bound on the energy constraint quantum capacity. Combining this with a new scheme of classical communication assistance, that combines forward and backward classical communication, they also obtain better lower bounds for the two-way classical communication assisted energy constraints quantum capacity. Finally, by constructing a classical-quantum-state, they provide a lower bound on the energy constraint private capacity that can outperform previous bounds.

As the authors point out in the discussion, it has already been shown in reference [52] that global encoding with correlated Gaussian states can result in a higher coherent information than product encoding. The significance of the present result is that it considers thermal loss channels with uncorrelated environmental noise, which are of greater practical relevance.

I believe the paper is interesting and well written, so it could be published. However the authors should explain in more detail how significant the difference is to the results presented in [52]. It might be clear to all the readers, why channels with uncorrelated environmental noise are of significantly greater relevance than correlated ones.

Reply–We thank the reviewer for the appreciation of our work and suggesting for us to elaborate more on the difference between our work and the results in Ref. [52] (which is now Ref. [53] in the revised manuscript). In the revised manuscript, we added a sentence (page 7, left column) explaining why the model with uncorrelated environmental thermal noise that we considered has greater practical relevance than the one with correlated noise.
